# Prenatal PBDEs and Neurodevelopment: Animal Studies and Human Health Assessment

**DOI:** 10.1289/ehp.1002753

**Published:** 2010-11

**Authors:** Marek Banasik

**Affiliations:** Institute of Public Health and Environmental Protection, Warsaw, Poland, E-mail: iphep.banasik@gmail.com

Herbstman et al. (2010) reported an association between polybrominated diphenyl ether (PBDE) levels in cord blood and neurodevelopmental effects in the children at specific ages. As a basis for their work, the authors cited several animal studies that reported causal relationships between prenatal exposure to PBDEs and developmental neurotoxicity. We are concerned that Herbstman et al.’s research suffers from investigator bias based on the reasons that follow.

First, the U.S. Environmental Protection Agency (EPA) cosponsored an expert panel that refuted the experimental design employed in most of the studies cited by Herbstman et al. (2010) as a basis for their work. The U.S. EPA expert panel concluded that the experimental design failed to control for litter effects (Holson et al. 2008).

Next, the potential for specific brominated flame retardants to cause developmental neurotoxicity has been evaluated under Good Laboratory Practice (GLP) standards and according to validated test guidelines. In each case, the claims of developmental neurotoxicity from non-GLP, non-guideline studies were not reproducible (reviewed by Williams and DeSesso 2010). This is significant because in Europe, data generated from studies performed under GLP and according to validated test guidelines are considered the highest quality and most reliable (European Chemicals Agency 2008). Further, regulatory agencies in Europe and the United States seem to have shifted their stance on the non-GLP, non-guideline studies that have reported brominated flame retardant–induced developmental neurotoxicity. For example, when the European Union issued their Risk Assessment Report on hexabromocyclododecane (HBCD), a brominated flame retardant (European Chemicals Bureau 2008), they stated that

… Eriksson et al. (2006) [i.e., the study reporting HBCD-induced developmental neurotoxicity] is not performed according to current guideline and GLP …. However, similar results on developmental neurotoxicity have been published for decabromodiphenylether by the same authors using the same method [e.g., Viberg et al. (2003), which was cited by Herbstman et al. (2010)]. For decabromodiphenylether it has been agreed to perform a new toxicokinetics/developmental neurotoxicity study according to a modified OECD guideline and GLP. The results from this new decabromodiphenylether study will serve as guidance on how to interpret the data from the Eriksson study, and may also serve as a basis on how to proceed with further testing of neurotoxicity.

For two of the studies cited by Herbstman et al. (2010), which were used by the U.S. EPA for deriving reference doses for PBDEs 153 and 209 (U.S. EPA 2008a, 2008b), the U.S. EPA was unable to obtain the raw data. However, when the raw data were obtained for the PBDE 209 study (i.e., Viberg et al. 2003) by a third party, who subsequently provided the data to the U.S. EPA, the agency acknowledged that the data were not suitable for use with human health assessment (U.S. EPA 2010).

We mention the above information because Herbstman et al. (2010) cited only animal studies that reported PBDE-induced developmental neurotoxicity as support for their work. Although the authors discussed one epidemiological study that reported findings inconsistent with their own, Herbstman et al. (2010) reverted back to the positive animal studies as support for their work. They did not discuss or cite any animal studies that reported contradictory findings. This is significant because it may have introduced a formidable source of bias when Herbstman et al. (2010) were interpreting their results. The exclusion may also mislead the readership of *EHP*.
